# *N*-Ethyl-*n*-Nitrosourea Induced Leukaemia in a Mouse Model through Upregulation of Vascular Endothelial Growth Factor and Evading Apoptosis

**DOI:** 10.3390/cancers12030678

**Published:** 2020-03-13

**Authors:** Abdullahi Aliyu, Mohd Rosly Shaari, Nurul Syahirah Ahmad Sayuti, Mohd Farhan Hanif Reduan, Shanmugavelu Sithambaram, Mustapha Mohamed Noordin, Khozirah Shaari, Hazilawati Hamzah

**Affiliations:** 1 Department of Veterinary Pathology and Microbiology, Faculty of Veterinary Medicine, Universiti Putra Malaysia UPM, Serdang 43400, Selangor, Malaysia; nurulsyahirah0806@gmail.com (N.S.A.S.); farhan.h@umk.edu.my (M.F.H.R.); noordinmm@upm.edu.my (M.M.N.); 2 Department of Veterinary Pathology, Faculty of Veterinary Medicine, City Campus Complex, Usmanu Danfodiyo University, 840212 Sokoto, Sokoto State, Nigeria; 3 Animal Science Research Centre, Malaysian Agricultural Research and Development Institute Headquarter, Serdang 43400, Selangor, Malaysia; rosly@mardi.gov.my (M.R.S.); shan.sithambaram@gmail.com (S.S.); 4 Department of Chemistry, Faculty of Science, Universiti Putra Malaysia UPM, Serdang 43400, Selangor, Malaysia; khozirah@upm.edu.my

**Keywords:** leukaemia, *n*-ethyl-*n*-nitrosourea, blast cells, angiogenesis, VEGF, apoptosis, BCL2

## Abstract

Chemical carcinogens are commonly used to investigate the biology and prognoses of various cancers. This study investigated the mechanism of leukaemogenic effects of *n*-ethyl-*n*-nitrosourea (ENU) in a mouse model. A total of 14 3-week-old male Institute of Cancer Research (ICR)-mice were used for the study. The mice were divided into groups A and B with seven mice each. Group A served as the control while group B received intraperitoneal (IP) injections of 80 mg/kg ENU twice with a one-week interval and were monitored monthly for 3 months for the development of leukaemia via blood smear examination. The mice were sacrificed humanely using a CO_2_ chamber. Blood, spleen, lymph nodes, liver, kidney and lung samples were collected for blood smear examination and histopathological evaluation. The expression of angiogenic protein (VEGF), and pro and anti-apoptotic proteins (BCL2 and BAX), was detected and quantified using Western blot technique. Leukaemia was confirmed by the presence of numerous blast cells in the peripheral blood smear in group B. Similarly, the VEGF and BCL2 proteins were significantly (*p* < 0.05) upregulated in group B compared to A. It is concluded that IP administration of 80 mg/kg ENU induced leukaemia in ICR-mice 12 weeks post administration through upregulation of angiogenic and anti-apoptotic proteins: VEGF and BCL2.

## 1. Introduction

Leukaemia refers to neoplastic proliferation of lymphoid and myeloid progenitor cells as a result of mutation of a single stem cell [1]. It is characterised by an abnormal accumulation of immature white blood cells (blast) in the blood and organs [2]. However, these blast cells are not able to perform the normal functions of blood cells [2], thereby causing increased susceptibility to infection. Leukaemia is broadly classified into acute and chronic leukaemia, and each is further subdivided into myeloid and lymphoid; i.e., acute myeloid leukaemia, acute lymphocytic leukaemia, chronic myeloid leukaemia, and chronic lymphocytic leukaemia [3]. Chronic lymphocytic leukaemia (CLL) has been reported to be the most common type of leukaemia found; however, acute myeloid leukaemia (AML) accounts for about 42% of all leukaemia deaths [4]. Leukaemia has been reported in humans and different species of animals, including horses, pigs, cats, cattle, mice, chickens and a variety of wild animals [5]. The number of neutrophils in acute leukaemic condition have been reported to be significantly lower [6]; meanwhile, those in chronic myeloid leukaemia (CML) exhibit defects in several functions [7].

Leukaemia, lymphoma and multiple myeloma are considered haematological cancers that affect mostly children, and adults in some instances [8]. These are liquid tumours moving in the blood and lymph. The causes of these malignancies are not well understood; however, some chemical carcinogens have been incriminated. Moreover, leukaemia has been reported to be induced by viruses, mutated genes and chemicals [4]. The disease is also associated with internal or external leukaemogenic factors, which cause chromosomal abnormalities resulting in DNA changes.

*N*-ethyl-*n*-nitrosourea (ENU) is an alkylating agent (chemical formula C3H7N3O2), that is a highly potent mutagen. It is reported to induce a single mutation in every 700 loci. The mechanism of action involves the transfer of alkyl (ethyl or methyl) group of ENU to the nucleobases in nucleic acids. The transfer of the alkyl group requires the action of an enzyme, alkyl transferase from the bone marrow; this causes decreased concentration of the enzymes within the bone marrow, leading to leukaemogenesis [9]. ENU has been largely used in several mutational studies [10]. It has been reported to induced recessive mutations consistently in mouse spermatogonia stem cells [11]. ENU and other nitrosoureas, including *n*-methyl-*n*-nitrosourea, were used extensively in the treatment of cancers in the past; they spontaneously decompose to generate two reactive species, i.e., an alkylating group and a carbamoylating group, both of which may react with DNA, RNA or protein, and they cause serious and often prolonged bone marrow suppression [12].

Angiogenesis involves the elaboration of the tubular network of endothelial cells, which is preceded embryogenically by vasculogenesis, where the endothelial cells differentiate to form mesodermal precursossors [13]. The process of angiogenesis continues throughout development, forming new capillaries through germinating or splitting from preformed vessels, followed by remodelling as the organism grows to adult stage. In adulthood, angiogenesis could be either physiological or associated with malignancies. The physiological and tumoural angiogenesis are controlled by host growth factors in the microenvironment, including vascular endothelial growth factor (VEGF), basic fibroblast growth factor (bFGF) and matrix metalloproteinases (MMPs). The majority of cancers studied have shown increased expression of VEGF, including haematological malignancies [14]; colon and rectal cancers [15]; liver cancers [16]; lung, breast, thyroid, gastrointestinal tract, kidney and bladder cancers; angiosarcomas; ovary and uterine cervix carcinomas; germ cell tumours; and intracranial tumours [17]. The growth and expansion of vascular supply is a critical factor in the development and metastatic spread of malignant tumours. Vascular endothelial growth factor (VEGF) is considered the most important growth factor in the regulation of physiological and pathological angiogenesis [13]. It has been reported that progression of haematolymphoid malignancies depends on the induction of new blood vessel formation under the influence of acute leukaemia, myelodysplastic syndromes, myeloproliferative neoplasms, multiple myeloma and lymphomas [18].

Apoptosis refers to programmed cell death, which is a phenomenon that differentiates between naturally occurring developmental cell death and cell death due to acute tissue injury, otherwise known as necrosis [19]. Apoptosis is responsible for the maintenance of tissue homeostasis, thereby ensuring an equilibrium between cell proliferation and cell death. Morphologically, apoptosis is characterised by blebbing of cell membrane, shrinkage of the apoptotic cells, chromatin condensation and nucleosomal fragmentation. The cells undergoing apoptosis are usually recognized and phagocytized by macrophages, or neighbouring cells that consume the cells’ fractionated carcasses.

There are two distinct molecular signalling pathways for apoptotic cell death: the intrinsic and extrinsic pathways. The former is considered the mitochondria-mediated pathway and is generally activated in response to intracellular stress signals, including DNA damage, high levels of reactive oxygen species (ROS), viral infection and activation of oncogenes, whereas the latter is the extracellularly mediated pathway, which is usually stimulated by binding of an extracellular ligand to a receptor on the plasma membrane [20]. Malignant diseases are associated with resistance to apoptosis, which is responsible for the unregulated proliferation of cells observed in many cancers [21].

Animal leukaemia model is very crucial in studying details of leukaemia progression and the treatment using natural products from plants against this debilitating disease [22,23]. This study is designed to investigate, in detail, the leukaemogenic effects of *n*-ethyl-*n*-nitrosourea in a mouse model.

## 2. Results

### 2.1. Weekly Bodyweight Gain

**Figure 1:**
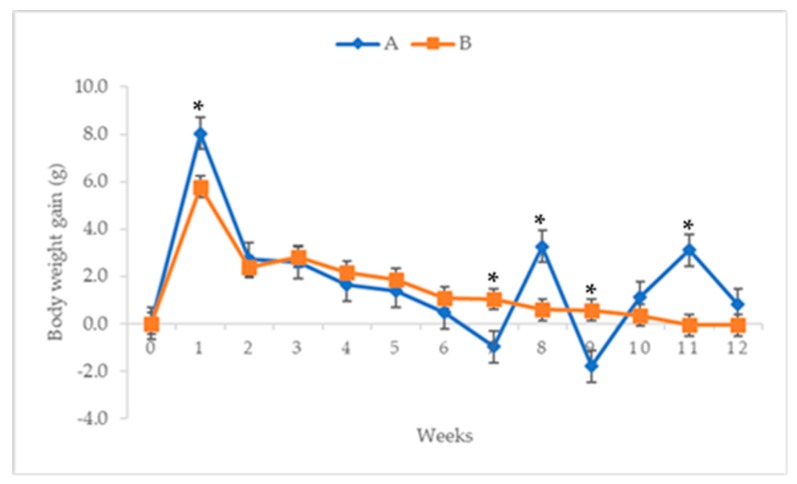
Average (mean ± SEM) weekly bodyweight gain (g) of male Institute of Cancer Research (ICR)-mice injected with 80 mg/kg *n*-ethyl-*n*-nitrosourea (ENU) twice at one-week interval. Key: A = control, B = treated with 80 mg/kg ENU twice with a 1-week interval, * significantly different at *p* < 0.05, SEM = standard error of mean.

The results of the bodyweight gains of the mice treated with ENU injections from week 0 to week 12 are presented on Figure 1. There were 28.0% and 12.1% reductions in the bodyweight gain of the mice in group B at weeks 1 (5.78 ± 0.96 g; *p* < 0.05) and 2 (2.40 ± 0.53 g; *p* > 0.05) of the experiment respectively, compared to group A at weeks 1 (8.04 ± 1.37 g) and 2 (2.72 ± 0.58 g) (Figure 1). However, these reductions were followed by 8.34%, 33.16%, 38.04%, 138.32%, 205.6% and 131.7% increases (*p* < 0.05) in the bodyweight gains of the mice in group B, at weeks 3, (2.82 ± 0.42 g; *p* > 0.05), 4 (2.17 ± 0.46 g; *p* > 0.05), 5 (1.88 ± 0.37 g; *p* > 0.05), 6 (1.09 ± 0.43 g; *p* > 0.05), 7 (1.03 ± 0.25 g; *p* < 0.05) and 9 (0.57 ± 0.35 g; *p* < 0.05) respectively, compared to group A, at weeks 3, (2.60 ± 0.24 g), 4 (1.63 ± 0.76 g), 5 (1.36 ± 0.17 g), 6 (0.46 ± 0.93 g), 7 (−0.98 ± 1.28 g) and 9 (−1.81 ± 1.39 g) (Figure 1). Moreover, the mice in group B presented 81.8%, 68.4%, 101.9% and 108.3% decreases in the average bodyweight gains at weeks 8 (0.59 ± 0.41 g; *p* < 0.05), 10 (0.35 ± 0.45 g; *p* > 0.05), 11 (−0.06 ± 0.34 g; *p* < 0.05) and 12 (−0.07 ± 0.84 g; *p* > 0.05) of the experiment respectively, compared to the mice in group A at weeks 8 (3.27 ± 1.13 g), 10 (1.11 ± 0.69 g), 11 (3.11 ± 1.07 g) and 12 (0.80 ± 0.23 g). (Figure 1).

### 2.2. Relative Organ Weights

**Figure 2:**
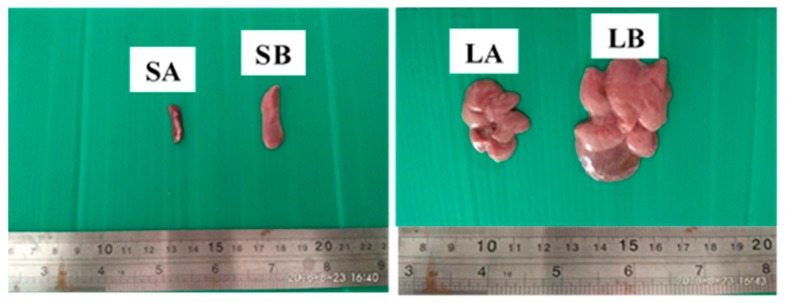
The effect of intraperitoneal (IP) injection of ENU on the gross appearance of spleen and liver of ICR-mice, 12 weeks post injection. Key: SA = normal spleen from a mouse in control group, SB = enlarged spleen from a mouse in ENU treated group showing splenomegaly, LA = normal liver from a mouse in control group, LB = enlarged liver from a mouse in ENU treated group showing hepatomegaly.

The effects of ENU injection on the relative organs’ weights of male ICR-mice were presented in Table 1. There was an 81.4%, significant (*p* < 0.05) increase in the relative organ weights of spleens (Figure 2(SB)) in group B (0.78 ± 0.26) compared to group A (0.43 ± 0.04). Moreover, the relative organ weights of lungs were 101% higher, significantly (*p* < 0.05), in group B (2.22 ± 0.63) compared to group A (1.10 ± 0.07). Similarly, the relative organ weight of the liver (Figure 2(LB)) was 33.8% higher (*p* > 0.05) in group B (6.30 ± 1.65) compared to A (4.71 ± 0.37). (Table 1).

### 2.3. Erythrogram Parameters of Leukaemia Induced Mice

The effects of IP injections of ENU on the erythrogram of male ICR-mice are shown on Table 2. There were no significant (*p* > 0.05) differences in the erythrogram parameters between groups A and B. However, the red blood cells were 8.8% higher (*p* > 0.05) in group B (10.31 ± 0.54 × 10^12^/L) compared to A (9.48 ± 0.33 × 10^12^/L). Similarly, the haemoglobin concentration was 7.9% higher (*p* > 0.05) in group B (157.33 ± 5.50 g/L) compared to A (145.80 ± 4.79 g/L) (Table 2). Alternatively, the packed cell volume was 41.4% lower (*p* > 0.05) in group B (0.58 ± 0.21 L/L) compared to A (0.99 ± 0.25 L/L). In addition, there was 6.9% decrease (*p* > 0.05) in the values of platelets in group B (1070.67 ± 244.18 × 10^9^) compared to those of A (1150.60 ± 257.02 × 10^9^) (Table 2).

### 2.4. Leukogram Parameters of Leukaemia Induced Mice

**Figure 3:**
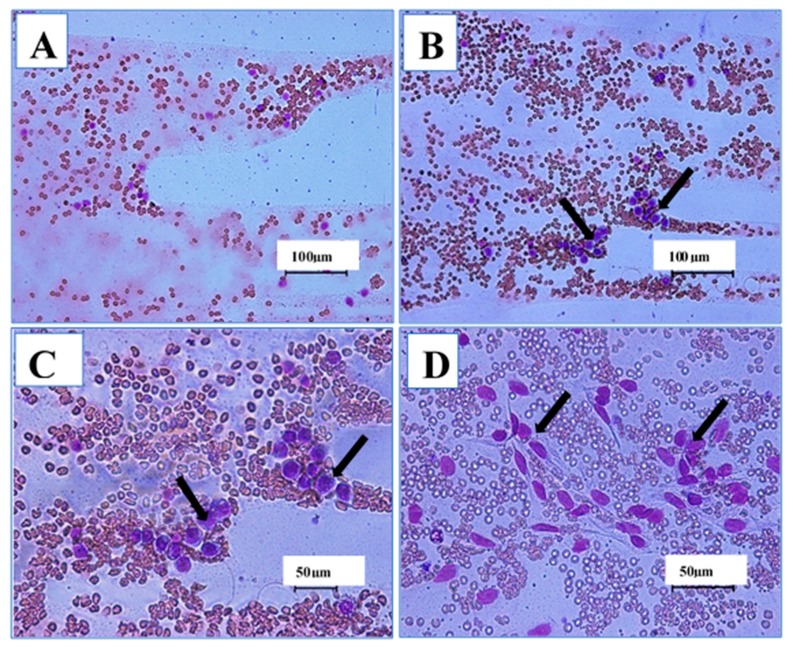
The effects of intraperitoneal (IP) injections of *n*-ethyl-*n*-nitrosourea (ENU) on the peripheral blood smears of male ICR-mice. Key: (**A**) Photomicrograph of blood smear (Wright stain ×100) from a mouse in control group showing normal morphology of white blood cells. (**B**) Photomicrograph of blood smear (Wright stain ×200) from a mouse in B group showing leukaemic blast cells (arrows) 12 weeks post IP injection of ENU. (**C**) Photomicrograph of blood smear (Wright stain ×400) from a mouse in B group showing leukaemic blast cells (arrows). (**D**) Photomicrograph of blood smear (Wright stain ×400) from a mouse in group B showing blast cells (arrows) presumably originated from bone marrow mesenchymal stromal cells (BM-MSCs). Scale bars are indicated at the respective images.

Table 3 shows the effects of IP injections of ENU on the leukogram of male ICR-mice 12 weeks post injection. Students’ “t” test showed 72.1% significant (*p* < 0.05) increase in total white blood cells in group B (13.32 ± 0.45 × 10^9^/L) compared to A (7.74 ± 0.95 × 10^9^/L). Correspondingly, the lymphocytes were also 42.1% higher in group B (7.19 ± 0.52 × 10^9^/L) compared to A (5.06 ± 0.70 × 10^9^/L) (Table 3). Furthermore, there was significant (*p* < 0.05) increase in the blast cells (Figure 3B–D) in group B (2.70 ± 0.73 × 10^9^/L) at week 12 compared to group A (0.00 ± 0.00 × 10^9^/L) (Table 3). Moreover, monocytes were 25.5% higher (*p* > 0.05) in group B (0.59 ± 0.10 × 10^9^/L) compared to A (0.47 ± 0.04 × 10^9^/L) Table 3.

### 2.5. Lesion Scoring

**Figure 4:**
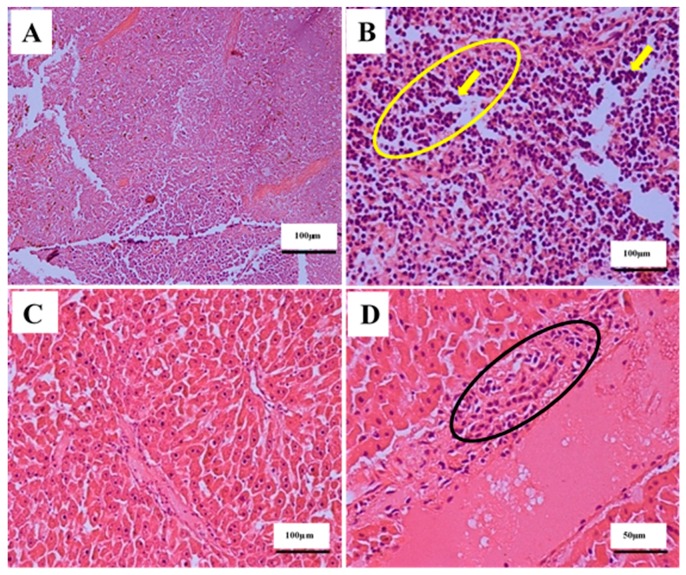
The effects of intraperitoneal (IP) injections of ENU on the histology of spleen and liver of ICR-mice, 12 weeks post injection. Key: (**A**) Photomicrograph of a spleen section (H&E stain ×200) from a mouse in control group showing normal histology of spleen. (**B**) Photomicrograph of a spleen section (H&E stain ×400) from a mouse in ENU treated group showing faded red pulp with numerous numbers of medium to large sized undifferentiated, bizarre pleomorphic nuclei (arrows) in the red pulp sinusoids and small focal aggregates in the splenic parenchyma (yellow encircled). (**C**) Photomicrograph of a liver section (H&E stain ×200) from a mouse in control group showing normal histology of liver. (**D**) Photomicrograph of a liver section (H&E stain ×400) from a mouse in ENU treated group showing metastasis of numerous numbers of medium to large size neoplastic lymphocytes to the liver (black encircled). Scale bars are indicated at the respective images. ENU = *n*-ethyl-*n*-nitrosourea, H&E = haematoxylin and eosin.

**Figure 5:**
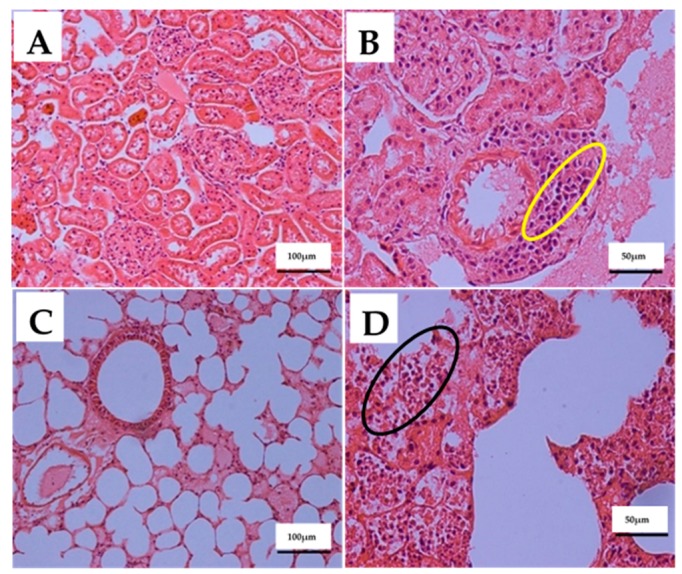
The effects of intraperitoneal (IP) injections of *n*-ethyl-*n*-nitrosourea (ENU) on the histology of kidney and lungs of ICR-mice, 12 weeks post injection. Key: (**A**) Photomicrograph of a kidney section (H&E stain ×200) from a mouse in control group showing normal histology of kidney. (**B**) Photomicrograph of a kidney section (H&E stain ×400) from a mouse in the ENU treated group showing clumps of medium to large size neoplastic lymphocytes infiltrated into the interstitial space of the renal tubules and around the renal artery (yellow encircled). (**C**) Photomicrograph of a lung section (H&E stain ×200) from a mouse in control group showing normal histology of lungs. (**D**) Photomicrograph of a lung section (H&E stain ×400) from a mouse in the ENU treated group showing clumps of medium to large size neoplastic lymphocytes around the pulmonary blood vessels and as focal aggregates in the lungs parenchyma, filling the alveolar spaces with enlarged alveolar septa (black encircled), H&E = haematoxylin and eosin. Scale bars are indicated at the respective images.

The effects of IP injections of 80 mg/kg ENU (twice at one-week interval) on the histology of spleen, liver, kidney and lung tissues of male ICR-mice are presented on Table 4. Mann–Whitney U test revealed significant (*p* < 0.05) differences in the lesion scores of the spleens, livers, kidneys and lungs between groups. There was mild (*p* < 0.05) increase in the proliferation of neoplastic lymphocytes in the spleens (Figure 4B) of mice in group B (1.33 ± 0.21) compared to A (0 ± 0.00) (Table 4). The neoplastic lymphocytes observed in the spleen were characterised by medium sized, undifferentiated, bizarre pleomorphic nuclei in the white and red pulps of the spleen (Figure 4B). Furthermore, the neoplastic lymphocytes appeared as individual cells in the red pulp sinusoids or as small focal aggregates in the splenic parenchyma (Figure 4B). Moreover, there was metastasis (*p* > 0.05) of small to large sized and aggregates of neoplastic lymphocytes around the central vein and hepatic sinusoids of the liver (Figure 4D); in interstitial space of the renal tubules; and around the renal artery (Figure 5B). Moreover, the neoplastic lymphocytes were also observed around the pulmonary blood vessels and as focal aggregates in the lungs’ parenchyma (Figure 5D) in group B compared to A (Table 4).

### 2.6. Terminal Deoxynucleotidyl Transferase dUTP Nick End Labelling (TUNEL) Apoptotic Assay

**Figure 6:**
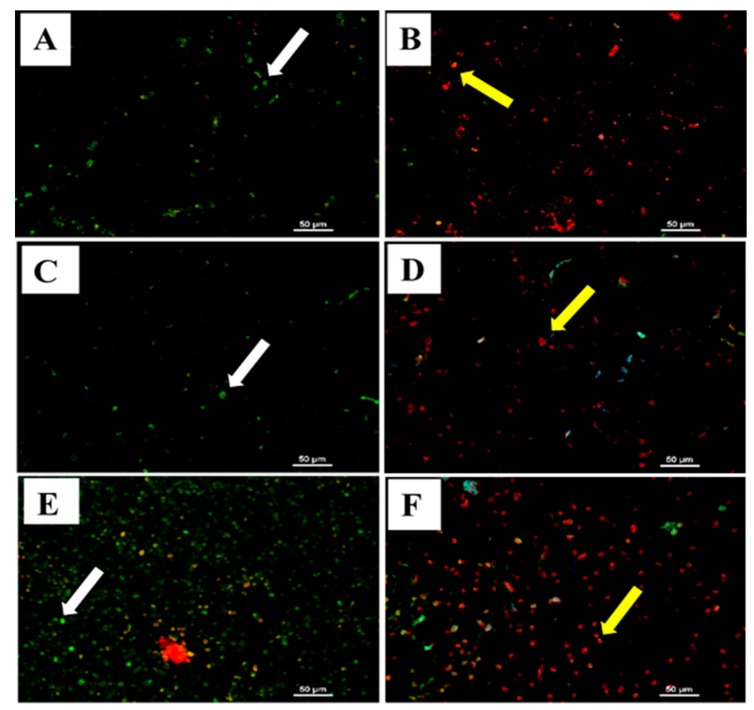
Detection of apoptosis using Terminal deoxynucleotidyl transferase dUTP nick end labelling (TUNEL) assay in the spleen, lymph nodes and liver tissues of male ICR-mice treated with 80 mg/kg *n*-ethyl-*n*-nitrosourea (ENU) twice at one-week interval. Key: (**A**) Photomicrograph of section of spleen from a mouse in group A showing few apoptotic cells (white arrow) in the spleen, characterised by green fluorescence (×400). (**B**) Photomicrograph of section of spleen from a mouse in group B showing numerous leukaemic cells (yellow arrow) in the spleens of ENU injected mice 12 weeks post injection; the leukaemic cells were characterised by red fluorescence (yellows arrow) when viewed under fluorescence microscope (×400). (**C**) Photomicrograph of section of lymph node from a mouse in group A showing few apoptotic cells (white arrow) in the lymph node, characterised by green fluorescence (×400). (**D**) Photomicrograph of section of lymph node from a mouse in group B showing numerous lymphoma cells (yellow arrow) in the lymph node of an ENU-injected mouse 12 weeks post injection; the lymphoma cells were characterised by red fluorescence (yellow arrow) when viewed under fluorescence microscope (×400). (**E**) Photomicrograph of section of liver from a mouse in group A showing few apoptotic cells (white arrow) in the liver of the mouse, characterised by green fluorescence (×400). (**F**) Photomicrograph of section of liver from a mouse in group B showing numerous leukaemic cells (yellow arrow) metastasised to the liver of ENU treated mice 12 weeks post injection; the metastasised leukaemic cells were characterised by red fluorescence (yellow arrows) when viewed under fluorescence microscope (×400). Scale bars are indicated at the respective images.

The effects of intraperitoneal injections of 80 mg/kg ENU (160 mg/kg total) on the incidence of apoptosis in the spleens, lymph nodes (submandibular and mesenteric) and livers of male ICR-mice are presented on Figure 6. There were numerous leukaemic cells in the spleen and lymph nodes (submandibular and mesenteric) of the male ICR-mice administered with IP injections of 80 mg/kg ENU 12 weeks post administration (Figure 6B,D), compared to group A (Figure 6A). These cells were characterised by red fluorescence when viewed under a fluorescence microscope (Figure 6B,D). Moreover, there were metastasis of the leukaemic cells into the liver in group B (Figure 6F) compared to A (Figure 6E), as evident by the prominent appearance of red fluorescence leukaemic cells under a fluorescence microscope (Figure 6F). However, the mice in group A showed few apoptotic cells in the spleen, lymph nodes and liver, characterised by green fluorescence (Figure 6A,C,E), when viewed under a fluorescence microscope, suggesting normal apoptotic processes going on in the spleens, lymph nodes (submandibular and mesenteric) and livers of the mice.

### 2.7. Western Blot for the Expression of VEGF, BCL2 and BAX Proteins in the Spleen and Lymph Nodes

**Figure 7:**
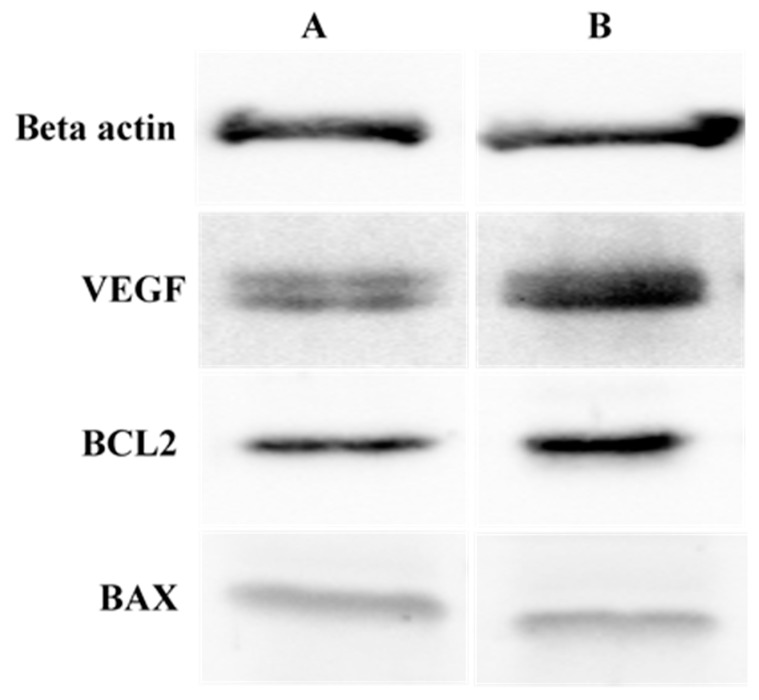
The effects of intraperitoneal (IP) injections of ENU on the expression of Beta actin, vascular endothelial growth factor (VEGF), B-cell lymphoma 2 (BCL2) and BCL2 associated X (BAX) in the spleens of male ICR-mice (representative samples). Key: ENU = *n*-ethyl-*n*-nitrosourea, A = control, B = treated with 80 mg/kg ENU twice with a 1-week interval.

The results of the Western blot analyses for the expression of VEGF, BCL2 and BAX in the spleens of male ICR-mice treated with 80 mg/kg doses of ENU are presented on Figure 7 and Table 5. The proteins beta actin, VEGF, BCL2 and BAX were all detected in both the control and the ENU treated groups (Figure 7). The results of the quantification of each of the protein bands revealed 248.6% significant (*p* < 0.05) upregulation in the expression level of VEGF in group B (7.53 ± 0.3) compared to A (2.16 ± 0.07) (Table 5). Similarly, the expression level of BCL2 was 280.2% higher (*p* < 0.05) in group B (4.41 ± 0.2) compared to A (1.61 ± 0.02) (Table 5). Moreover, there was 273.8% significant (*p* <0.05) fold increase in the expression level of BAX in group B (2.28 ± 0.10) compared to A (0.610 ± 0.02) (Table 5).

## 3. Discussion

Leukaemia refers to the cancer of blood-forming cells in the bone marrow characterised by an abnormal increase in the immature white blood cells otherwise known as blast cells [1]. The blast cells accumulate in the blood and organs, but cannot perform their normal defensive functions, thereby causing increased susceptibility to infection [2]. Leukaemia is one of the most devastating haematologic cancers; in recent years, the disease accounted for about 8% of all cancers in adults, and the incidence of the disease increases with age [8,25,26]. Aliyu et al. [8] reported that the exact causes of leukaemia are not properly understood; nevertheless, certain factors, including alkylating drugs, chemicals and ionising radiation, have been incriminated to have induced chromosomal abnormalities leading to DNA damage [27]. Nitrosoureas, including *n*-ethyl-*n*-nitrosourea (ENU), can induce leukaemia by transferring an alkyl group to a nucleobase, thereby causing DNA changes leading to serious, mostly prolonged bone marrow suppression and subsequent leukaemogenesis [8,9,28]. ENU is considered a potent carcinogenic agent [29,30] that can induce several cancers in animal models, including brain tumours, reproductive tumours, leukaemia and others [31]. The chemical has previously been successfully used to induce leukaemia in mouse models [32,33,34,35]. The process of blood cell formation from single stem/progenitor cells is constantly under continuous stimulation of various factors secreted from its surrounding accessory cells [8]. Any modification in the signalling cascade of differentiation and proliferation leads to haematopoietic disorders which may either be accelerative or decelerative in nature. This may lead to the normal haematopoietic cells been transformed into leukaemic cells, thereby shifting the balance between cell proliferation and cell death, resulting in a cumulative increase of leukaemic cells within bone marrow and peripheral blood [36]. The cellular mechanism through which ENU induces leukaemia in animals may be associated with its carcinogenicity [37] or its immunosuppressive effects [38,39].

The continuous decrease in the average bodyweight gain observed in this study, in the ENU treated mice, suggests that the chemical might have affected the appetites of the treated mice, or the leukaemic burden might have affected the metabolisms of the treated mice [40,41,42]. This is in agreement with the report of Chang et al. [3], where the average bodyweight of NMU-treated Sprague Dawley (SD) rats was less than that of the control group. The significant increase in the relative organ weights of spleen and lungs observed in the mice treated with ENU could be due to infiltration or metastasis of leukaemic cells into these vital organs [43]. This is similar to the report of Chang et al. [3], wherein the livers and spleens of leukaemic rats induced by NMU were enlarged due to invasion of leukaemic cells.

Furthermore, the significant increase in the total white blood cells (WBC), lymphocytes, monocytes and neutrophils and the significant increase in the blast cells at week 12 of the experiment in the ENU treated group compared to untreated control group might indicate active leukaemic condition due to the injection of the chemical ENU in the treated mice. This is because leukaemia is characterised by abnormal or impaired differentiation of haematopoietic stem cells, resulting in an abnormal accumulation of immature precursors and a suppression of growth and maturation of cells involved in normal hemopoiesis [44]. A significant increase in WBC with a decrease in relative neutrophils was reported previously [3]. Similarly, Akanni et al. [45] reported that the mean WBC and number of lymphocytes of leukaemic rats induced with benzene carcinogen were significantly higher than those of the control group. The findings of this research were supported further by the report of Law et al. [32], where intraperitoneal administration of ENU induced a mixed type of leukaemia 4–7 months post injection; the leukaemia was predominantly lymphoblastic in nature. Furthermore, mice challenged with ENU injection by Bhattacharjee et al. [34] were reported to develop leukaemia five months post injection; the leukaemia was confirmed by the appearance of numerous blast cells in peripheral blood and bone marrow smears; and a significant increase in total leukocyte counts in the ENU challenged group compared to the control was observed [34]. The results reported by Singha et al. [35] were also in agreement with those of this study, where there were numerous blast cells in the peripheral blood smears from ENU-treated mice compared to the control group, suggesting active leukaemic condition in the former group of mice [35].

The TUNEL apoptotic assay revealed numerous red fluorescence leukaemic cells in the spleens and lymph nodes of the mice that received IP injections of total of 160 mg/kg ENU compared to the control group, which showed green fluorescence cells. Furthermore, the protein analysis by Western blot revealed that the group of mice treated with the injections of ENU in this study had significant upregulation of angiogenic protein VEGF and anti-apoptotic protein BCL2 compared to the control group, signifying leukaemogenic and anti-apoptotic activities in the treated mice [18]. The significant upregulation in the expression levels of VEGF could suggest that administration of ENU induces leukaemia in the treated mice [46]. This is because VEGF has been reported as one of the most important angiogenesis stimulating factors, which contributes to the cancer progression through its tumour neovascularisation, tumour invasion, metastasis and resistance to chemotherapy [46,47,48]. Moreover, previous studies have shown that over expression of VEGF has been associated with various types of cancers, including haematological malignancies [14]; colon and rectal cancers [15]; liver cancers [16]; and lung, breast, thyroid, gastrointestinal tract, kidney and bladder cancers [17]. Moreover, the upregulation in the expression of anti-apoptotic protein BCL2 in this study could suggest that ENU induced leukaemia in mice model through evading apoptosis [49,50]. Apoptosis has been reported as a hallmark for various cancer types, including leukaemia, and overexpression of anti-apoptotic BCL2 protein plays an important role in the regulation of apoptosis [51,52]. In addition, overexpression of antiapoptotic protein BCL2 has been reported as a requirement for the genesis of cancer and resistance of cancer cells to apoptosis [21].

## 4. Materials and Methods

### 4.1. Animal Handling

The experiment was conducted at the Animal Metabolism, Toxicology and Reproductive Centre (AMTREC), Malaysian Agricultural Research and Development Institute (MARDI), Serdang. This research adhered to the guide for the care and use of laboratory animals and was approved by the Animal Ethics Committee (AEC) of MARDI with the approval reference number: 20170717/R/MAEC00024. The mice were acclimatized for 1 week to the housing conditions, with temperature within the range of 22–25 °C, humidity at the range of 40–70% and balance of 12 h light/12 h dark cycle. The bedding and water were replaced regularly, and the cages cleaned accordingly. Each mouse was placed in a polycarbonate plastic cage. The mice were weighed on day 0 (before the administration of the chemical) and then repeatedly, weekly, throughout the study periods.

### 4.2. Determination of Weekly Bodyweight and Bodyweight Gain

The bodyweight of each mouse in each of the experimental groups was measured weekly using an electric weighing scale and recorded as described elsewhere [53]. The weekly bodyweight gain was calculated by subtracting the previous weekly bodyweight of each mouse from that of the current week.

### 4.3. Experimental Design

A total of 14 3-week-old male ICR-mice were acclimatized for 1 week and grouped into 2 groups, A and B, with 7 mice each. Group A served as control and was given normal saline, while group B was given IP injection of 80 mg/kg twice with a one-week interval [35].

### 4.4. N-Ethyl-n-Nitrosourea (ENU) Preparation and Administration

The *n*-ethyl-*n*-nitrosourea (ENU) was purchased from Sigma Aldrich (St. Louis, MO, USA). It was stored at −20 °C until needed for the experiment. The chemical carcinogen was prepared freshly on each of the administration day by dissolving the required quantity according to the average bodyweight of the mice in the treatment group in the required volume of normal saline. The dissolved powder was mixed thoroughly by vortex to dissolve the solute properly. Eighty (80) mg/kg bodyweight of the dissolved powder was injected to each of the mice in the treatment group via intraperitoneal route using an insulin syringe with a 26-gauge needle. The mice were injected twice with the same dose at one-week interval [35]. The mice were closely monitored for any signs of toxicity and/or mortality. Feed and water were made available for the mice ad libitum.

### 4.5. Haematology Analysis

Blood samples were collected at week 12 of the experiment for blood smear examination and for the complete blood count. The procedure was as described below:

The blood samples collected were analysed for complete blood count using an automated haematology analyser (ABC Vet ®, ABX Diagnostics, Montpellier, France) for the total red blood cells (RBC), WBC, platelet count, haemoglobin (Hb) concentration, mean corpuscular volume (MCV), mean corpuscular haemoglobin concentration (MCHC) and differential leukocyte counts. Blood smears were prepared and stained with Wright stain and examined under a light microscope [53,54,55].

### 4.6. Histopathology Analysis

Selected organs, including the spleen, the lymph nodes, the liver, the kidney and the lungs were collected from each mouse for histopathological analyses. The procedure for analyses was according to Nurul et al. [54] as described below:

Liver and kidneys were collected from each mouse at the end of the experiment and cleaned using cold normal saline to remove excess blood from the tissues. Collected organs were fixed in 10% neutral buffered formalin, which was changed after 24 h for better fixation. The organs were then further processed at the Histopathology Laboratory, Faculty of Veterinary Medicine, Universiti Putra Malaysia. The samples were trimmed at about 0.5 cm thickness and placed in histological cassettes for tissue processing. The cassettes (with the tissues) were immersed in 10% formalin overnight and then transferred into an automated processor (ASP300, Leica Biosystems, Nussloch, Germany) for 16 h to undergo series of dehydration processes. The samples were then embedded in paraffin to form a block using a processor machine (EG1160, Leica Biosystems) and trimmed to about 3–5 µm thickness using a sectioning rotary microtome (RM2155, Leica Biosystems) and directly placed the tissue sections into a 45 °C water bath prior to being mounted on clean glass slides. The glass slides were labelled appropriately using a pen and placed on a hot plate (54 °C) overnight for proper mounting of the tissues on the slides. The slides were then stained with haematoxylin and eosin (H&E) and examined under light microscope at various magnifications including 40, 100, 200 and 400. The microscopic analysis of the stained tissues was carried out blindly, and any histopathological changes deviant from the norm were carefully noted and recorded.

#### Lesion Scoring Characteristics

The histopathological lesions were observed and scored for the spleens and lymph nodes of both control group and the leukaemia-bearing mice in group B. The lesions were also evaluated in other vital organs, including the liver, kidney and lungs to check for possible metastasis; the details of the scoring are shown on the Table 6, Table 7 and Table 8 below.

### 4.7. TUNEL Apoptotic Assay

The incidence of apoptosis was evaluated using “In situ BrdU-Red DNA Fragmentation (TUNEL) assay Kit” (ab6110 Abcam, Cambridge, MA, USA) according to the manufacturer’s instructions. The kit is a convenient and sensitive method to detect DNA fragmentation by fluorescence microscopy. The kit uses BrdUTP (bromolated deoxyuridine triphosphate nucleotide), which can be incorporated into DNA strand breaks; the greater incorporation rate produces a brighter signal when the BrdUTP sites are detected with an anti BrdU monoclonal antibody directly labelled with a red fluorochrome. The details of the procedure were as described below [56,57].

#### 4.7.1. Histopathological Preparations

Spleen, lymph node and liver tissues were collected from each mouse at the end of the experiment and cleaned using cold normal saline to remove excess blood from the tissues. The tissues were processed as described in Section 4.6, except that the slides were not subjected to H&E staining protocol.

#### 4.7.2. Deparaffinization and Dehydration Protocol

The slides were immersed in a Coplin jar containing fresh xylene and incubated two times for 5 min each at room temperature to remove the paraffin. The slides were then immersed in 100% ethanol and incubated for 5 min at room temperature. The slides were further rehydrated by sequential 3-minute, room temperature incubations in Coplin jars containing 100%, 95%, 85%, 70% and 50% ethanol respectively. The slides were then immersed in Coplin jar containing 0.85% NaCl and incubated for 5 min at room temperature. Thereafter, the slides were washed two times in PBS for 5 min each at room temperature. Fluids were allowed to drain thoroughly, and the slides were placed on a flat surface.

One millilitre of 20 µg/mL Proteinase K solution (2 µL Proteinase K 10 mg/mL + 998 µL Tris-HCl pH 8.0 + 50 mM EDTA) was prepared, and each tissue section was covered with 100 µL of the solution and incubated for 5 min at room temperature, and then washed in PBS for 5 min at room temperature. The slides were transferred to a Coplin jar containing 4% formaldehyde in PBS and incubated for 5 min at room temperature. The slides were washed in PBS for 5 min at room temperature.

#### 4.7.3. Labelling

The slides were removed from PBS and the excess liquid was gently removed. The tissue section was covered with 100 µL of wash buffer and a piece of plastic cover slip was gently placed on top of the tissue section to evenly spread the liquid, and it was incubated for 5 min at room temperature. The coverslip was carefully removed, and the slide was tapped gently to remove excess liquid and the edges were blotted dry with tissue paper. Each slide was covered with 50 µL of DNA labelling solution and a piece of plastic cover slip was gently placed on top of the tissue section to evenly spread the liquid, and the slides were incubated in a dark humidified incubator for 1 hour at 37 °C. The plastic coverslip was gently removed, and the slides were rinsed two times in PBS for 5 min each; the edges were blotted dry with tissue paper. The slides were then covered with 100 µL of antibody solution; then a piece of plastic cover slip was gently placed on top of the tissue section to evenly spread the liquid, and the slides were incubated in a dark for 30 min at room temperature.

#### 4.7.4. Detection by Fluorescence Microscopy

The slides were washed in ddH_2_O for 5 min at room temperature and 100 µL of 7-AAD/RNase A staining buffer was added to each of the slides to counter stain the DNA. A piece of plastic cover slip was gently placed on top of the tissue section to evenly spread the liquid and the slides were incubated in a dark for 30 min at room temperature. The slides were washed in ddH_2_O two times for 5 min each at room temperature and were blotted dry with tissue paper. The tissue sections were analysed by fluorescence microscope (Nicon eclipse Ti, Leica, New York, NY, USA) at Ex/Em = 488/576 nm (BrU-Red) and Ex/Em = 488/655 nm (if using 7AAD).

### 4.8. Analysis of Protein Expression

#### 4.8.1. Protein Extraction from the Spleen

The total proteins from the spleen of each experimental mouse was extracted using T-PER ® Tissue Protein Extraction Reagent (Thermo Scientific, Waltham, MA, USA). The protease and phosphatase inhibitor cocktail was added to the T-PER ® Tissue Protein Extraction Reagent at the ratio of 1:9 to inhibit serine protease, aminopeptidase-B, leucine aminopeptidase, cysteine protease and metalloproteases, and at the same time to inhibit phosphatases, which include serine/threonine phosphatases, acid phosphatases, protein tyrosine phosphatases and alkaline phosphatases.

The spleen tissue was weight and T-PER was added into a clean tube at the ratio of 0.05 g of tissue to 1 mL of T-PER reagent and homogenised using a micro pestle. The sample was then centrifuged at 10,000 × *g* for 5 min using micro-refrigerated centrifuge to pellet the tissue debris. The supernatant was carefully collected into a new tube and stored at −80 °C for downstream analyses.

#### 4.8.2. Bicinchonic Acid (BCA) Assay

The total protein concentration in each of the spleen samples was determined using Pierce™ BCA Protein Assay kit (Thermo Scientific, Walthan, MA, USA). The protein detection range of the kit is 20 to 2000 µg/mL. Standard and working reagents were prepared accordingly. The albumin standard (BSA) was diluted into several clean vials using T-PER^®^ Tissue Protein Extraction Reagent (Thermo Scientific, Waltham, MA, USA) (Table 9).

The working reagent was prepared according to the formula below. Total volume of working reagent = (number of standards + unknown) × (replicates) × (volume or WR per well).

The BCA working reagent was prepared by mixing BCA reagent A with BCA reagent B at the ratio of 50:1. On addition of BCA reagent B to A, turbidity was observed, which quickly disappeared upon mixing, yielding a clear green working reagent.

The micro plate procedure of the Pierce™ BCA protein assay kit was employed. About 25 µL each of the standard and unknown protein sample replicate were pipetted into a 96-well micro plate (Tecno Plastic products 96-well plate, Trasadingen, Switzerland). About 200 µL of BCA working reagent was added into each well containing standards and samples at the ratio of 1:8. The micro plate was mixed thoroughly on an orbital shaker (Rotamax 120 Orbital shaker, Heidolph, Schwabach, Germany) for 30 s. The plate was covered and incubated at 37 °C for 30 min. The plate was cooled down to room temperature and the absorbance was measured at 562 nm on a plate reader (Infinite 200 PRO TECAN, Mannedorf, Switzerland). Blank-corrected absorbance of each individual standard and sample replicate was calculated by subtracting the average absorbance measurement of the blank sample from the absorbance measurements of each individual standard and sample replicates. Standard curve was plotted using excel and was used to determine the protein concentration of each of the unknown samples.

#### 4.8.3. Protein Lysis

About 300 µg of protein from spleen sample from each of the treatments and control groups was diluted with sample buffer, Laemmli 2× concentration (Sigma Aldrich, St. Louis, MO, USA, SLBT2606) at the ratio of 1:1; the mixture was diluted further by sample buffer Laemmli 1× concentration to meet up with the required uniform volume in all the samples. The protein samples were heated at 97 °C using Corning LSE digital dry bath (Sigma Aldrich, St. Louis, MO, USA) for 5 min and centrifuged using refrigerated microcentrifuge at 16,000 × *g* for 5 min. The samples were stored at −20 °C for long term storage.

#### 4.8.4. Western Blot Analysis

About 40 µg protein samples from the spleens of each of the mouse in both the control and the ENU treated groups were loaded into separate lanes of 10% sodium dodecyl sulphate-polyacrylamide gel electrophoresis (SDS-PAGE). The gel was run for 60 min at 100V and later for 45 min at 150 V to separate the proteins by electrophoresis. The gel was then placed onto a Trans-Blot Turbo Mini polyvinylidene fluoride (PVDF) membrane (Bio-Rad, Hercules, CA, USA) to transfer the separated proteins; any air bubbles trapped on the membrane were removed using a roller. The transfer was run for 150 min at 20 V using Trans-Blot Turbo Transfer System (Bio-rad, Hercules, CA, USA). The PVDF membrane was then washed with TBST (Tris buffered saline tween 20 (0.12 M Tris-base, 1.5 M NaCl, 0.5% Tween-20, pH 7.6)) for 5 minutes at room temperature on a shaker before been blocked with 5% non-fat milk in TBST for 1 h at room temperature on a shaker. The membrane was later incubated with the appropriate primary antibody (rabbit monoclonal to VEGF (Abcam, Cambridge, UK, ab32152) (1:1000), rabbit polyclonal to BCL2 (Abcam, UK, Cn 59348) (1:500), rabbit polyclonal to BAX (Abcam, UK, Cn 7977) (1:500) and rabbit polyclonal to Beta actin (Abcam, UK, ab8227) (1:2000), Abcam, Cambridge, MA, USA) in 3% blocking buffer overnight at 4 °C on a shaker. The PVDF membrane was washed with TBST three times for 5 min each at room temperature on a shaker before been incubated with 1:2000 dilution of secondary antibody (Goat Anti-Rabbit IgG H&L (HRP) (Abcam, UK, ab205718)) in 3% blocking buffer for 1 h at room temperature on a shaker. The membrane was washed again with TBST three times for 5 min each at room temperature on a shaker. The proteins of interest were detected using Radiance Plus^TM^ enhanced chemiluminescence (ECL) femtogram HRP substrate (Azure Bioscience, Dublin, CA, USA). The signals (bands) were viewed and captured using gel documentation system (GBOX-CHEMI-HR1-4, Syngene, Frederick, MD, USA).

The expression of each of the proteins analysed was normalised by beta actin and then quantified by dividing the intensity of each of the proteins from all the treatment group with that of the control group. The whole blots showing expression level of beta actin, VEGF, BCL2 and BAX proteins can be found at Supplementary (Figures S1–S4).

### 4.9. Statistical Analyses

The results of the relative organ weights, haematology and quantitative Western blot analyses were expressed as mean ± SEM (standard error of mean) in tables, and the differences between the means of the two groups were analysed using student’s “t” statistical tool using IBM SPSS statistical software version 23. The results of lesion scoring were analysed using non-parametric Mann–Whitney U test using the same SPSS software version 23. Statistically significant differences were set at *p* < 0.05.

## 5. Conclusions

This study concluded that intraperitoneal administrations of ENU at 80 mg/kg bodyweight twice with a one week interval induced leukaemia in ICR-mice 12 weeks post administration, as evident by the remarkable increase in the organ to bodyweight of the spleen; significant increase in the leukaemic blast cells, WBC and lymphocyte counts; and notable increase in the proliferation of neoplastic lymphocytes in the spleen and other vital organs in the treated mice. Furthermore, this study has demonstrated the leukaemogenic effects of ENU administration in ICR-mice through up regulation of the angiogenic protein: VEGF. The study has demonstrated further that the chemical carcinogen exerts its leukaemic effect through evading apoptosis; this is true because of the remarkable upsurge of the anti-apoptotic protein, the BCL2 in the ENU treated group of mice, compared to the control.

## Figures and Tables

**Table 1:** The effects of ENU on relative organs’ weights in % (mean ± SEM) of male ICR-mice.

Organs (%)	A	B
Liver	4.71 ± 0.37	6.30 ± 1.65
Right Kidney	0.79 ± 0.06	0.73 ± 0.06
Left Kidney	0.75 ± 0.06	0.71 ± 0.04
Spleen	0.43 ± 0.04	0.78 ± 0.26 *
Heart	0.50 ± 0.04	0.56 ± 0.03
Lungs	1.10 ± 0.07	2.22 ± 0.63 *
Testes	0.67 ± 0.04	0.61 ± 0.03

Note: number of determinants (n) in each group = 7, ENU = *n*-ethyl-*n*-nitrosourea, A = control, B = treated with 80 mg/kg ENU twice with a 1-week interval, values in the same row with asterisk differ significantly (*p* < 0.05).

**Table 2:** Haematogram (mean ± SEM) of ENU-induced, leukaemic, male ICR-mice.

Parameters	A	B	Reference ^a^
Red blood cells (×10^12^/L)	9.48 ± 0.33	10.31 ± 0.54	8.49–10.43
Haemoglobin (g/L)	145.80 ± 4.79	157.33 ± 5.50	138–168
PCV (L/L)	0.99 ± 0.25	0.58 ± 0.21	0.47–0.529
Platelets (×10^9^)	1150.60 ± 257.02	1070.67 ± 244.18	784–1812
MCV (fl)	54.80 ± 0.87	55.17 ± 0.54	42.3–48
MCH (pg)	15.38 ± 0.18	15.37 ± 0.39	14.7–17.5
MCHC (g/L)	280.40 ± 3.19	278.67 ± 4.86	341–367
Plasma proteins (g/L)	80.80 ± 4.34	82.00 ± 3.83	-

Note: number of determinants (n) in each group = 7, ENU = *n*-ethyl-*n*-nitrosourea, A = control, B = treated with 80 mg/kg ENU twice with a 1-week interval, PCV = packed cell volume, MCV = mean corpuscular volume, MCH = mean corpuscular haemoglobin, MCHC = mean corpuscular haemoglobin concentration, ^a^ = Serfilippi et al. [24], values in the same row without asterisk are comparable (*p* > 0.05).

**Table 3:** Leukogram (mean ± SEM) of ENU-induced, leukaemic, male ICR-mice.

Parameters	A	B	Reference ^a^
White blood cells (×10^9^/L)	7.74 ± 0.95	13.32 ± 0.45 *	7.06–17.29
Neutrophils (×10^9^/L)	2.19 ± 0.24	2.84 ± 0.44	0.98–6.06
Lymphocytes (×10^9^/L)	5.06 ± 0.70	7.19 ± 0.52 *	5.01–11.6
Monocytes (×10^9^/L)	0.47 ± 0.04	0.59 ± 0.10	0.09–0.63
Eosinophils (×10^9^/L)	0.02 ± 0.02	0.00 ± 0.00	0–0.75
Basophils (×10^9^/L)	0.00 ± 0.00	0.00 ± 0.00	0–0.09
Blast cells (×10^9^/L)	0.00 ± 0.00	2.70 ± 0.73 *	-

Note: number of determinants (n) in each group = 7, ENU = *n*-ethyl-*n*-nitrosourea, A = control, B = treated with 80 mg/kg ENU twice with a 1-week interval, ^a^ = Serfilippi et al. [24], values in the same row with asterisk differ significantly (*p* < 0.05).

**Table 4:** Histological lesion scoring (mean ± SEM) for leukaemia in the spleen, liver, kidney and lung tissues of ENU treated mice, 12 weeks post inoculation.

Organ	Histopathological Observations	A	B
Spleen	Increased proliferation of neoplastic lymphocytes	0 ± 0.00	1.33 ± 0.21 *
Liver	Metastasis of malignant lymphocytes in the hepatic sinusoids	0 ± 0.00	0.17 ± 0.17
Kidney	Metastasis of malignant lymphocytes in the glomeruli and renal tubules	0 ± 0.00	0.33 ± 0.21
Lungs	Metastasis of malignant lymphocytes in the lung parenchyma	0 ± 0.00	0.83 ± 0.40

Note: number of determinants (n) in each group = 7, ENU = *n*-ethyl-*n*-nitrosourea, A = control, B = treated with 80 mg/kg ENU twice with a 1-week interval, * = values in the same row with asterisk differ significantly (*p* < 0.05).

**Table 5:** Actin normalised values for relative expression of VEGF, BCL2 and BAX in ENU injected male ICR-mice.

Protein	A	B
VEGF	2.16 ± 0.07	7.53 ± 0.3 *
BCL2	1.61 ± 0.02	4.41 ± 0.2 *
BAX	0.610 ± 0.02	2.28 ± 0.10 *

Note: number of determinants (n) in each group = 3, VEGF = vascular endothelial growth factor, BCL2 = B-cell lymphoma 2, BAX = BCL2 associated X, ENU = *n*-ethyl-*n*-nitrosourea, A = control, B = treated with 80 mg/kg ENU twice with a 1-week interval, values in the same row with asterisk differ significantly (*p* < 0.05).

**Table 6:** Histopathological lesion scoring in the spleen of leukaemia-bearing mice.

Area	Score	Criteria
Red pulp	0	Normal histology
	1	Small size of clusters of small size neoplastic lymphocytes
	2	Large size of clusters of small to medium size neoplastic lymphocytes. Pleomorphism of neoplastic lymphocytes.
	3	Diffused proliferation of medium to large size neoplastic lymphocytes. Pleomorphism of neoplastic lymphocytes. Diminished architecture of red pulp of spleen
White pulp	0	Normal histology
	1	Small size of clusters of small size neoplastic lymphocytes
	2	Large size of clusters of small to medium size neoplastic lymphocytes. Pleomorphism of neoplastic lymphocytes.
	3	Diffused proliferation of medium to large size neoplastic lymphocytes. Pleomorphism of neoplastic lymphocytes. Diminished architecture of white pulp of spleen

**Table 7:** Histopathological lesion scoring in the lymph nodes of leukaemia bearing mice.

Organ	Score	Criteria
	0	Normal histology
Lymph nodes	1	Small neoplastic lymphocytes in cortical areas
	2	Small to medium size of neoplastic lymphocytes in cortical and paracortical areas. Pleomorphism of neoplastic lymphocytes.
	3	Medium to large size of neoplastic lymphocytes in cortical, paracortical and medullary areas. Pleomorphism of neoplastic lymphocytes. Diminished architecture of red pulp of spleen

**Table 8:** Histopathological lesion scoring in the liver, kidneys, and lungs of leukaemia-bearing mice.

Organ	Score	Criteria
Liver	0	Normal Histology
	1	A few numbers of metastasized small size neoplastic lymphocytes
	2	Moderate number of metastasized small to medium size neoplastic lymphocytes. Dilated hepatic sinusoids infiltrated with neoplastic lymphocytes. Pleomorphism of neoplastic lymphocytes.
	3	Numerous numbers of metastasized medium to large size neoplastic lymphocytes. Diminished architecture of liver tissue Pleomorphism of neoplastic lymphocytes.
Lungs	0	Normal Histology
	1	A few numbers of metastasized small size neoplastic lymphocytes
	2	Moderate number of metastasized small to medium size neoplastic lymphocytes.
	3	Numerous numbers of metastasized medium to large size neoplastic lymphocytes. Diminished architecture of lungs tissue Pleomorphism of neoplastic lymphocytes.
Kidneys	0	Normal Histology
	1	A few numbers of metastasized small size neoplastic lymphocytes
	2	Moderate number of metastasized small to medium size neoplastic lymphocytes.
	3	Numerous numbers of metastasized medium to large size neoplastic lymphocytes. Diminished architecture of kidney tissue Pleomorphism of neoplastic lymphocytes.

**Table 9:** Preparation of albumin standards for the BCA assay.

Tube	Volume of T-PER (µL)	Volume of Albumin BSA (µL)	Final BSA Conc. (µg/(µL)
A	0	300 stock	2000
B	125	375 stock	1500
C	325	325 stock	1000
D	175	175 of tube B	750
E	325	325 of tube C	500
F	325	325 of tube E	250
G	325	325 of tube F	125
H	400	100 of tube G	25
I	400	0	0

## Supplementary Materials

The following are available online at https://www.mdpi.com/2072-6694/12/3/678/s1, Figure S1: Western blot for the analyses of the expression level of beta actin protein in the spleen of ICR-mice injected with 80 mg/kg ENU (n-ethyl-n-nitrosourea), Figure S2: Western blot for the analyses of the expression level of VEGF protein in the spleen of ICR-mice injected with 80 mg/kg ENU (n-ethyl-n-nitrosourea), Figure S3: Western blot for the analyses of the expression level of BCL2 protein in the spleen of ICR-mice injected with 80 mg/kg ENU (n-ethyl-n-nitrosourea), Figure S4: Western blot for the analyses of the expression level of BAX protein in the spleen of ICR-mice injected with 80 mg/kg ENU (n-ethyl-n-nitrosourea).
