# Supplementary material for: N-Ethyl-n-Nitrosourea Induced Leukaemia in a Mouse Model through Upregulation of Vascular Endothelial Growth Factor and Evading Apoptosis
Source: Cancers (Basel). 2020 Mar 13;12(3):678. doi: 10.3390/cancers12030678 (PMC7140055; doi:10.3390/cancers12030678)
Supplement: Supplementary file 1 [file cancers-12-00678-s001.pdf]

Supplementary materials

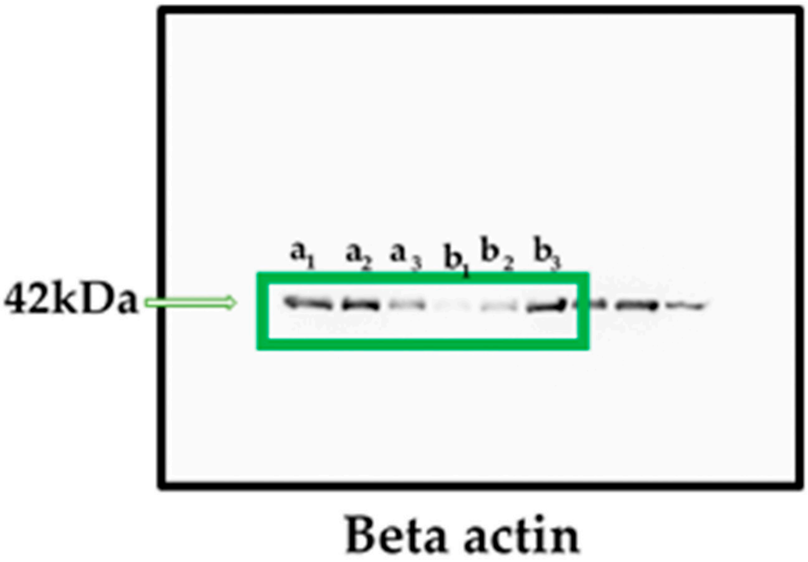

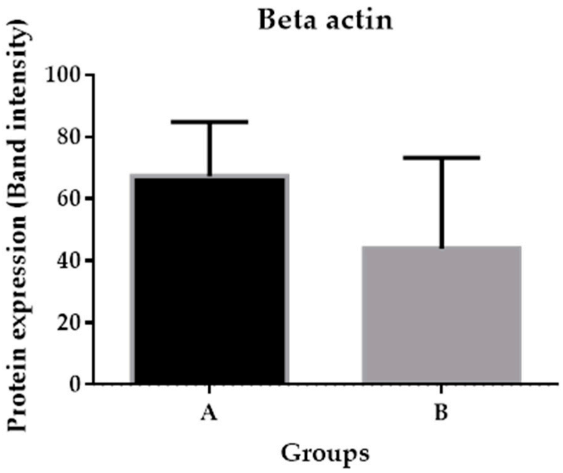

Figure S1. Western blot for the analyses of the expression level of beta actin protein in the spleen of male ICR-mice injected with 80 mg/kg ENU (*n*-ethyl-*n*-nitrosourea). Key: a_1-a_3 = samples from control group treated with normal saline, b_1-b_3 = samples from group B treated with 80 mg/kg ENU.

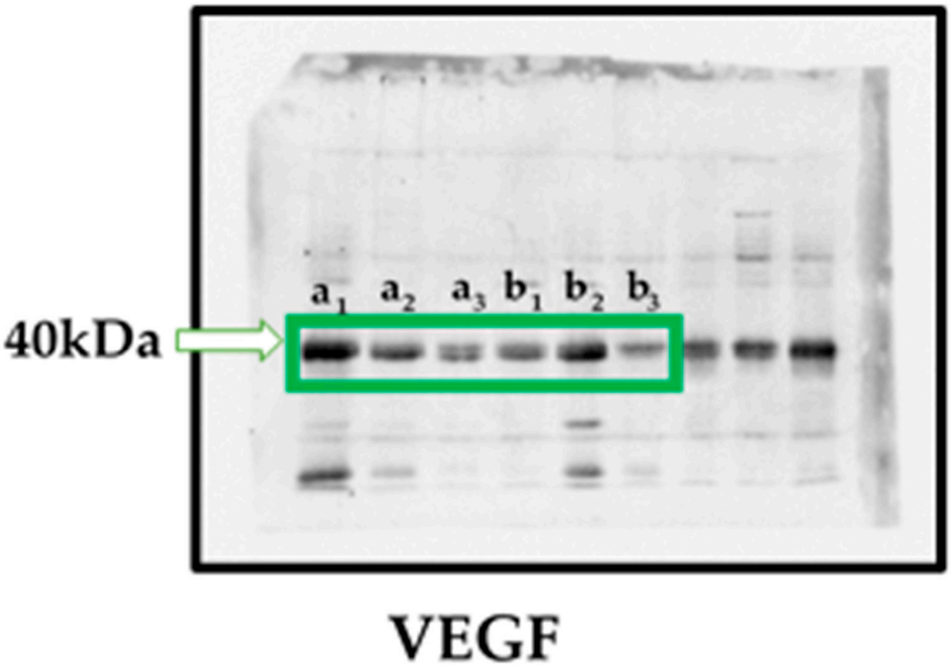

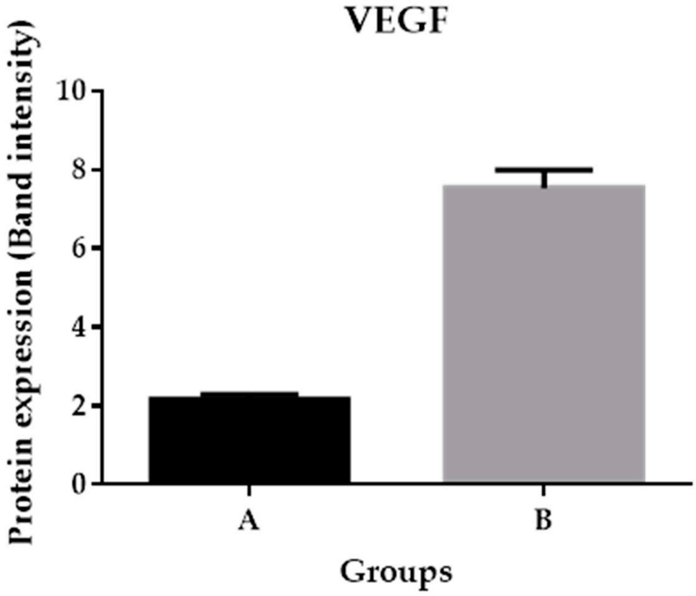

Figure S2. Western blot for the analyses of the expression level of VEGF protein in the spleen of male ICR-mice injected with 80 mg/kg ENU (*n*-ethyl-*n*-nitrosourea). Key: a_1-a_3 = samples from control group treated with normal saline, b_1-b_3 = samples from group B treated with 80 mg/kg ENU.

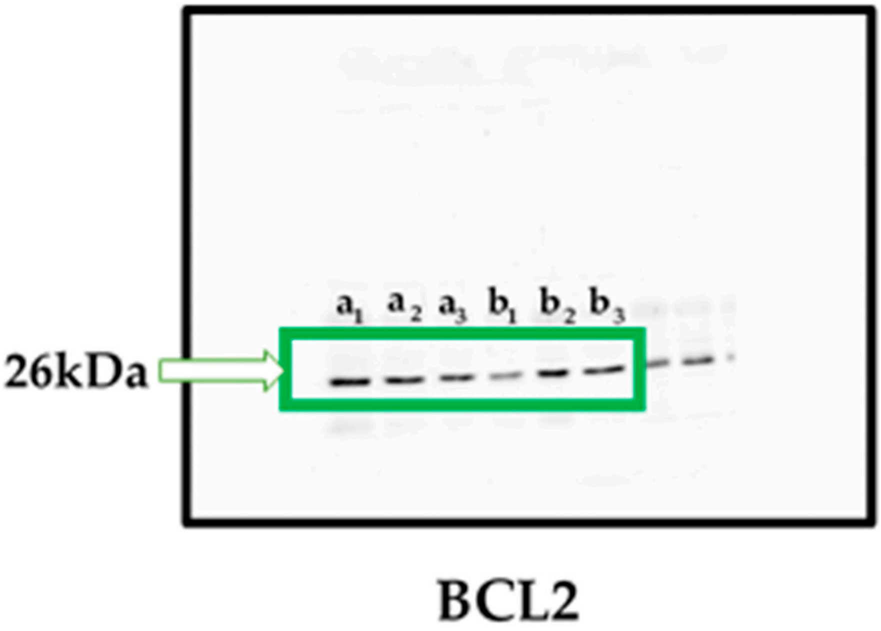

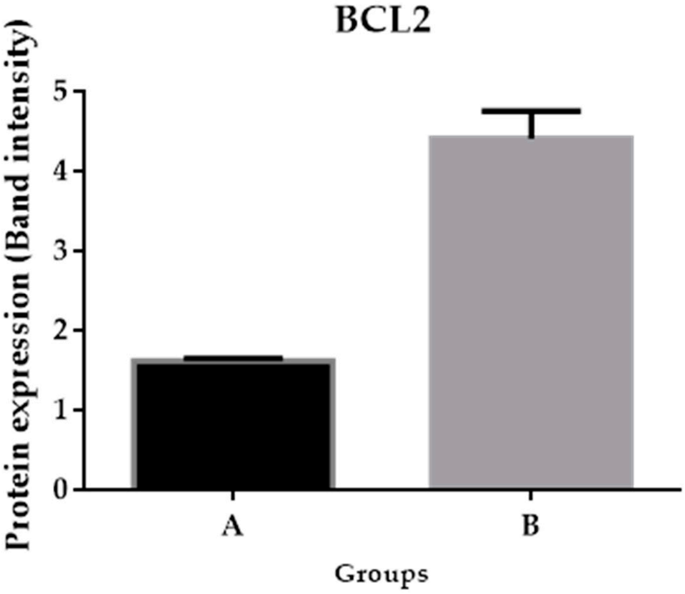

Figure S3: Western blot for the analyses of the expression level of BCL2 protein in the spleen of male ICR-mice injected with 80 mg/kg ENU(*n*-ethyl-*n*-nitrosourea). Key: a_1-a_3 = samples from control group treated with normal saline, b_1-b_3 = samples from group B treated with 80 mg/kg ENU.

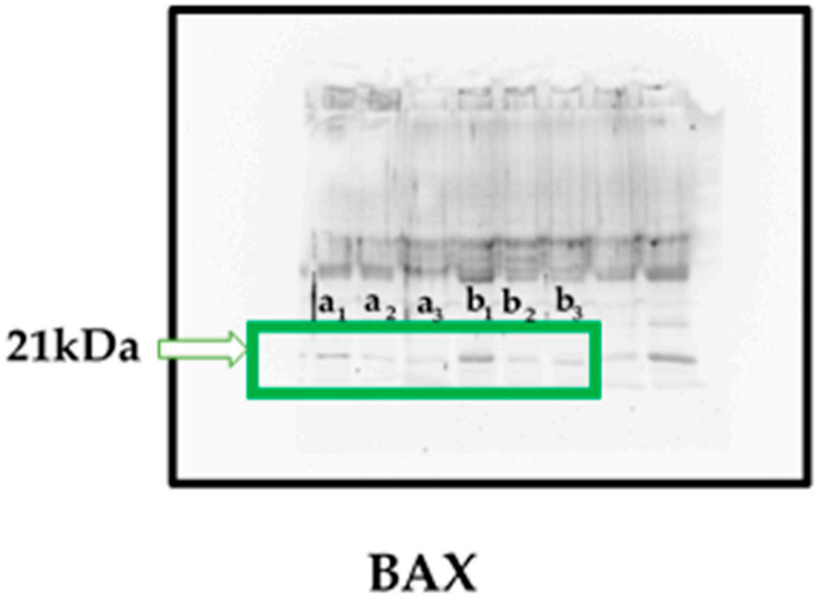

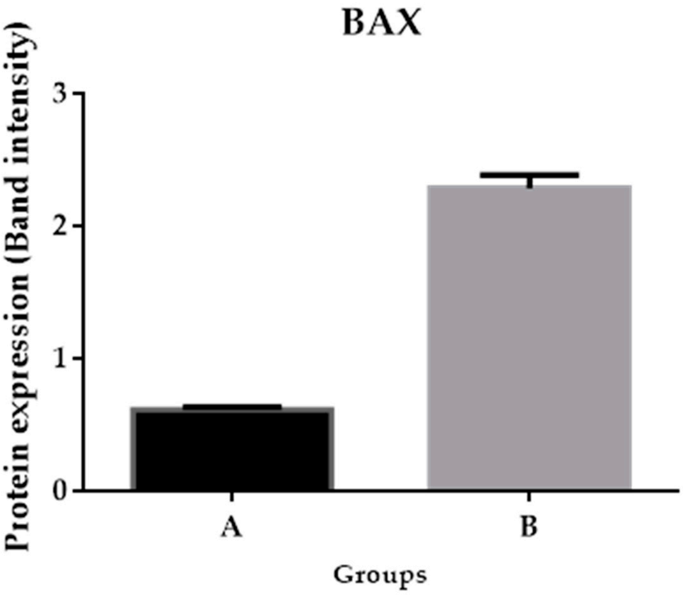

Figure S4: Western blot for the analyses of the expression level of BAX protein in the spleen of male ICR-mice injected with 80 mg/kg ENU (*n*-ethyl-*n*-nitrosourea). Key: a_1-a_3 = samples from control group treated with normal saline, b_1-b_3 = samples from group B treated with 80 mg/kg ENU.
